# Information Needs in the Precision Medicine Era: How Genetics Home Reference Can Help

**DOI:** 10.2196/ijmr.5199

**Published:** 2016-04-27

**Authors:** Heather Collins, Sherri Calvo, Kathleen Greenberg, Lisa Forman Neall, Stephanie Morrison

**Affiliations:** ^1^ ICF International Fairfax, VA United States; ^2^ National Institutes of Health US National Library of Medicine Bethesda, MD United States; ^3^ Medical Science & Computing, LLC Rockville, MD United States

**Keywords:** individualized medicine, patient education as topic, databases, genetic, health resources

## Abstract

Precision medicine focuses on understanding individual variability in disease prevention, care, and treatment. The Precision Medicine Initiative, launched by President Obama in early 2015, aims to bring this approach to all areas of health care. However, few consumer-friendly resources exist for the public to learn about precision medicine and the conditions that could be affected by this approach to care. Genetics Home Reference, a website from the US National Library of Medicine, seeks to support precision medicine education by providing the public with summaries of genetic conditions and their associated genes, as well as information about issues related to precision medicine such as disease risk and pharmacogenomics. With the advance of precision medicine, consumer-focused resources like Genetics Home Reference can be foundational in providing context for public understanding of the increasing amount of data that will become available.

## Introduction

In January 2015, President Obama announced the Precision Medicine Initiative [1], a research effort aimed at changing how diseases are diagnosed and treated in the United States. Its goal is to bring precision medicine—an approach to disease management that considers individual variability in genes, environment, and lifestyle—into the field of cancer and then ultimately into all areas of health and health care [2,3]. Integral to the initiative’s effectiveness is the recruitment of a longitudinal cohort of 1 million volunteers, who will be overseen by the National Institutes of Health (NIH). This cohort will provide genetic data, biological samples, and other health information to researchers [4]. Detailed analyses of this database of health information, including genome-sequencing data and lifestyle and environmental factors, will help determine the genetic contribution to disease development, identify disease risk factors, and generate effective treatments that incorporate the way genes affect a person’s response to drugs (a field of study called pharmacogenomics). This knowledge will enable clinicians to use genetic and other molecular information as part of routine medical care.

Reliable resources are necessary to support the public as they seek to educate themselves. Individuals searching online for information about the Precision Medicine Initiative may encounter unfamiliar concepts relating to health and genetics. Genetics Home Reference [5], an online resource from the US National Library of Medicine, provides consumer-focused information on various topics related to precision medicine, including how genetic variants relate to disease, pharmacogenomics, and genetic testing. In the era of precision medicine, online resources aimed at the general public, like Genetics Home Reference, are needed as more people become interested in the genetic aspects of health care.

## Information Needs in the Precision Medicine Era

The need for new tools for researchers and clinicians to store, manage, and analyze large amounts of data has been discussed as a key factor in the implementation and success of precision medicine [2,6,7]. While storage and management of these data will be challenging, current tools may be helpful for data analysis. For example, to parse the meaning of newly identified genetic changes, researchers and clinicians can use tools such as Polymorphism Phenotyping (PolyPhen) [8] to determine whether a genetic change is likely involved in the development of disease. For pharmacogenomics associations, researchers and clinicians can use Pharmacogenomics Knowledgebase (PharmGKB) [9], a database that organizes information about genetic variants playing a role in drug response. Resources such as these will expand as more data are collected through precision medicine, making the databases more robust and increasingly useful for analysis.

While the utility of clinical resources for precision medicine has so far been paramount, the need for patient resources is equally important. Resources that are accessible for health care consumers can be used as a starting point for understanding precision medicine and its applications to health care. The influx of data generated by precision medicine means individuals will have access to more details about their health than ever before when making precision medicine-based health care decisions [10]. For people to make informed decisions in the era of precision medicine, it is imperative that they have an understanding of basic genetic principles; however, studies suggest that a substantial proportion of the general public lacks this understanding [11-13]. To support patient engagement in precision medicine and promote informed decision making, both clinicians and patients will require trusted online resources that provide easy-to-read information about genetic principles, genetic disorders, gene functions and their roles in disease, and pharmacogenomics. The use of Internet-based health tools increases patient engagement, which leads to better health outcomes [14].

The Internet is a major tool people use to research their health concerns; up to 80% of adults on the Internet report searching for health-related topics annually [15,16]. As precision medicine is adopted in clinical settings, it is inevitable that the public will turn to the Internet for information, as they have for other health inquiries. The Genetic and Rare Disease Information Center, an NIH resource that provides information targeted to consumers, reports that their users are primarily looking for diagnosis, prognosis, and treatment information for particular diseases [17]. A customer satisfaction survey on Genetics Home Reference also suggests that this website’s users are looking for information related to precision medicine. Our staff is collecting results from an ongoing survey of randomly selected website users, who are provided with the opportunity to suggest improvements via open-ended questions. The survey’s unpublished, preliminary results indicate a strong interest in the relationship between genetic mutations and disease course, the role of genetics in treatment options, and the interaction of lifestyle and genetic factors in disease. Survey respondents want information that is applicable to their particular health situation, with one user lamenting that “There is very limited information on my specific mutation.” Another user stated their interest as simply wanting to know “about mutations in real life.” (unpublished data, 2015). While conclusions cannot be drawn from this qualitative data, these responses do suggest that Genetics Home Reference users have an interest in precision medicine and the application of genetic information.

Clinicians can also benefit from patient resources; clinicians can add to their own knowledge and share these resources with patients. Continuing education of health care professionals is vital to the long-term success of the Precision Medicine Initiative [18]. Despite the spread of genetic testing into various areas of medicine, many clinicians lack familiarity with genetics and the important role it plays in health care. Recent reports from the Secretary’s Advisory Committee on Genetics, Health, and Society [19] and the Secretary’s Advisory Committee on Heritable Disorders in Newborns and Children [20] raise concerns about the amount of medical genetics education health care workers receive. Health care professionals need an understanding of genetic concepts to interpret precision medicine data and explain them to patients. As a supplement to professional training, point-of-care education allows the clinician to become knowledgeable in specific topics as they present in practice [21]. In addition, clinicians can use consumer resources to help explain genetic conditions and concepts to their patients.

## Genetics Home Reference and Precision Medicine

Genetics Home Reference provides information targeted to patients and families with genetic disorders and to individuals interested in genetics who do not have a science background. Genetics Home Reference receives an average of 1.5 million visitors and 3.6 million page views each month. This website offers summaries of more than 1000 genetic conditions and more than 1300 genes. To construct these summaries, pertinent information is gleaned from scientific literature and written into summaries using language that can be understood by the lay public. Genetics Home Reference has information on dozens of topics aimed at educating the public about issues related to precision medicine, including genetic risk factors for disease and pharmacogenomics. For example, this resource provides information about the function of the *BRCA1* and *BRCA2* genes and explains how a mutation in either of these genes increases the risk of developing breast cancer and other types of cancer [22]. The presence of a mutation in either of these genes can help determine appropriate cancer screening and treatment approaches. Genetics Home Reference also offers information about genetic factors that alter a person’s response to a common blood-thinning drug called warfarin. These genetic variants predispose people who might need warfarin to develop either blood clots (warfarin resistance) [23] or abnormal bleeding (warfarin sensitivity) [24]. If a patient had one of these genetic variants, a doctor might target the initial warfarin dose for optimum effectiveness and reduce the risk of an adverse drug reaction.

Genetics Home Reference also covers other types of cancer, immune deficiencies and dysfunctions, enzyme deficiencies, and other drug sensitivities (see Table 1). A benefit to clinicians is the inclusion of numerous rare conditions that might never be covered during formal education, in addition to a variety of common disorders.

**Table 1:** A sample of conditions on Genetics Home Reference to which precision medicine could be applied.

Condition		Genetics Home Reference link
**Cancers**		
	Breast cancer	https://ghr.nlm.nih.gov/condition/breast-cancer
	Lynch syndrome	https://ghr.nlm.nih.gov/condition/lynch-syndrome
	Prostate cancer	https://ghr.nlm.nih.gov/condition/prostate-cancer
	Familial adenomatous polyposis	https://ghr.nlm.nih.gov/condition/familial-adenomatous-polyposis
	Acute promyelocytic leukemia	https://ghr.nlm.nih.gov/condition/acute-promyelocytic-leukemia
	Neuroblastoma	https://ghr.nlm.nih.gov/condition/neuroblastoma
	Core binding factor acute myeloid leukemia	https://ghr.nlm.nih.gov/condition/core-binding-factor-acute-myeloid-leukemia
**Immune system disorders**		
	Celiac disease	https://ghr.nlm.nih.gov/condition/celiac-disease
	Type 1 diabetes	https://ghr.nlm.nih.gov/condition/type-1-diabetes
	Autoimmune Addison disease	https://ghr.nlm.nih.gov/condition/autoimmune-addison-disease
	Rheumatoid arthritis	https://ghr.nlm.nih.gov/condition/autoimmune-addison-disease
	Graves disease	https://ghr.nlm.nih.gov/condition/graves-disease
	Autoimmune lymphoproliferative syndrome	https://ghr.nlm.nih.gov/condition/amyotrophic-lateral-sclerosis
	Systemic lupus erythematosus	https://ghr.nlm.nih.gov/condition/systemic-lupus-erythematosus
**Enzyme deficiencies**		
	Lactose intolerance	https://ghr.nlm.nih.gov/condition/lactose-intolerance
	Glucose-6-phosphate dehydrogenase deficiency	https://ghr.nlm.nih.gov/condition/glucose-6-phosphate-dehydrogenase-deficiency
	Hereditary antithrombin deficiency	https://ghr.nlm.nih.gov/condition/hereditary-antithrombin-deficiency
	Familial hypercholesterolemia	https://ghr.nlm.nih.gov/condition/hypercholesterolemia
	Protein C deficiency	https://ghr.nlm.nih.gov/condition/protein-c-deficiency
	Autosomal recessive congenital methemoglobinemia	https://ghr.nlm.nih.gov/condition/autosomal-recessive-congenital-methemoglobinemia
	Gaucher disease	https://ghr.nlm.nih.gov/condition/gaucher-disease
**Adverse drug reactions**		
	Warfarin sensitivity	https://ghr.nlm.nih.gov/condition/warfarin-sensitivity
	Warfarin resistance	https://ghr.nlm.nih.gov/condition/warfarin-resistance
	Malignant hyperthermia	https://ghr.nlm.nih.gov/condition/malignant-hyperthermia
	Pseudocholinesterase deficiency	https://ghr.nlm.nih.gov/condition/pseudocholinesterase-deficiency
	Dihydropyrimidinase deficiency	https://ghr.nlm.nih.gov/condition/dihydropyrimidinase-deficiency
	Thiopurine S-methyltransferase deficiency	https://ghr.nlm.nih.gov/condition/thiopurine-s-methyltransferase-deficiency
	Dihydropyrimidine dehydrogenase deficiency	https://ghr.nlm.nih.gov/condition/dihydropyrimidine-dehydrogenase-deficiency
	Stevens-Johnson syndrome/toxic epidermal necrolysis	https://ghr.nlm.nih.gov/condition/stevens-johnson-syndrome-toxic-epidermal-necrolysis

Genetics Home Reference also provides a primer called *Help Me Understand Genetics* for individuals who need foundational information. This primer has multiple chapters, covering topics from basic biology to the application of genetics in medicine. The precision medicine section of the primer explains this new approach to health care as well as the goals, benefits, and limitations of the Precision Medicine Initiative (see Table 2). Also of interest are health care–based issues, such as chapters on mutations and health, pharmacogenomics, and genetic testing. *Help Me Understand Genetics* provides information on the many facets of genetic testing that individuals will need to become familiar with as genetic testing becomes more routine for disease diagnosis and defining treatment options, such as indications for testing, interpretation of test results, and the difference between research and clinical testing.

**Table 2:** Background information about precision medicine from Genetics Home Reference.

Topic	Genetics Home Reference link
What is precision medicine?	https://ghr.nlm.nih.gov/handbook/precisionmedicine/definition
What is the difference between precision medicine and personalized medicine? What about pharmacogenomics?	https://ghr.nlm.nih.gov/handbook/precisionmedicine/precisionvspersonalized
What is the Precision Medicine Initiative?	https://ghr.nlm.nih.gov/handbook/precisionmedicine/initiative
What are some potential benefits of precision medicine and the Precision Medicine Initiative?	https://ghr.nlm.nih.gov/handbook/precisionmedicine/potentialbenefits
What are some of the challenges facing precision medicine and the Precision Medicine Initiative?	https://ghr.nlm.nih.gov/handbook/precisionmedicine/challenges
What is pharmacogenomics?	https://ghr.nlm.nih.gov/handbook/genomicresearch/pharmacogenomics

### Conclusion

Consumers are required to take an increasingly active role in their health care decisions, and they turn to the Internet to gather information regarding health issues. In the era of precision medicine, individuals will search for information to understand their genetic profiles and other health concerns. Various aspects of precision medicine are covered in online resources, including ClinVar [25], Genetic Testing Registry (GTR) [26], GeneReviews [27], Online Mendelian Inheritance in Man (OMIM) [28], and Orphanet [29]. However, these resources are designed primarily for researchers and clinicians, using technical information and language that can be overwhelming for most lay individuals.

Few consumer-focused resources about precision medicine exist, and the need for such resources will only increase. Resources are needed to put into context the growing amount of genetic and other health data that are becoming available [5]. Genetics Home Reference provides consumer-friendly information on topics relevant to precision medicine, including genetic conditions, gene function, and the effects of genetic variation on health, genetic testing, and pharmacogenomics. This information is useful to a variety of people, patients and clinicians alike, as the public increasingly turns to the Internet as a health resource. During this era of precision medicine, Genetics Home Reference seeks to facilitate health consumers in becoming well informed.

Research into consumers’ specific information needs related to precision medicine could help guide the evolution of existing educational resources and the development of new resources [30]. These studies should first assess existing resources to identify areas that are not covered. For example, we are unaware of any comprehensive consumer databases that connect specific genetic variants to the development of disease or that outline treatment options based on particular genetic profiles. Such resources would help patients gather targeted information that is specific to their health situation and would be valuable additions in the age of precision medicine.

Genetics Home Reference is committed to supporting patient engagement through the ongoing addition of new information that is relevant to precision medicine. To determine which topics are relevant for inclusion on the website, Genetics Home Reference staff frequently collaborates with outside groups that include other NIH institutes, advocacy and support groups, and unsolicited user feedback. These collaborations ensure that the content on Genetics Home Reference appeals to a wide range of audiences. Genetics Home Reference will also continue to provide links to other reputable online resources that offer information beyond our scope. Developers of consumer-focused health resources, including Genetics Home Reference, have the opportunity to be proactive in providing education about precision medicine to the public concurrently with the implementation of this new approach to care.

## Abbreviations

GTR: Genetic Testing Registry
NIH: National Institutes of Health
OMIM: Online Mendelian Inheritance in Man
PharmGKB: Pharmacogenomics Knowledgebase
PolyPhen: Polymorphism Phenotyping
